# IL-6, IL-1RA and Resistin as Predictors of Left Ventricular Remodelling and Major Adverse Cardiac Events in Patients with Acute ST Elevation Myocardial Infarction

**DOI:** 10.3390/diagnostics12020266

**Published:** 2022-01-21

**Authors:** Alina Ioana Scărlătescu, Miruna Mihaela Micheu, Nicoleta Popa-Fotea, Ana Maria Pascal, Ana Maria Mihail, Ioana Petre, Silvia Deaconu, Aura Vîjîiac, Maria Dorobanțu

**Affiliations:** 1 Department of Cardiology, “Carol Davila” University of Medicine and Pharmacy, 050474 Bucharest, Romania; alina.scarlatescu@gmail.com (A.I.S.); fotea.nicoleta@yahoo.com (N.P.-F.); i_comanescu@yahoo.com (I.P.); aura.apostolescu@yahoo.com (A.V.); maria.dorobantu@gmail.com (M.D.); 2 Emergency Clinical Hospital Bucharest, Department of Cardiology, 014461 Bucharest, Romania; annaa_pas@yahoo.com (A.M.P.); mihailanamaria94@yahoo.ro (A.M.M.); silvia.iancovici@yahoo.com (S.D.)

**Keywords:** STEMI, cytokines, LVR, MACE

## Abstract

Despite continuous advances in diagnostic and therapeutic methods, acute myocardial infarction (AMI) remains a leading cause of morbidity and mortality worldwide. Considering the role of inflammation in AMI etiopathogenesis, we aimed to explore the role of a group of three inflammatory cytokines (IL-1RA, IL-6 and resistin) as an independent prognostic factor for LVR assessed by 3D echocardiography and MACE in patients with STEMI. We enrolled 41 patients with STEMI who underwent primary PCI. We assessed the occurrence of LVR (defined as an increase of over 20% in end-diastolic left ventricular volume at 6 months compared with baseline values) and MACE. Using the enzyme-linked immunosorbent assays (ELISA) method, we measured plasmatic levels of IL-6, IL-1RA and resistin (within 48 h after AMI and at 6 months). Out of 41 STEMI patients, 20.5% presented signs of LVR at follow up, and in 24.4%, MACE occurred. In univariate logistic regression analysis, baseline levels of IL-6 (OR = 1.042, *p* = 0.004), IL-1RA (OR = 1.004, *p* = 0.05) and resistin (OR = 1.7, *p* = 0.007) were all significantly associated with LVR. ROC analysis showed that the three cytokines as a group (AUC 0.946, *p* = 0.000) have a better predictive value for LVR than any individual cytokine. The group of cytokines also proved to have a better predictive value for MACE together than separately (AUC = 0.875, *p* = 0.000 for ROC regression model). IL-6, IL-1RA and resistin plasma levels at baseline have a good predictive value both as independent variables and also as a group for the development of adverse LVR and MACE at 6 months follow up after STEMI.

## 1. Introduction

The prognosis of STEMI is mainly determined by the extent of irreversible myocardial injury and LVR. After AMI, the left ventricle undergoes a series of histopathological and structural changes that can lead to adverse LVR [1]. It is a complex process that involves both the infarcted and non-infarcted myocardium, leading to changes in shape, size and function of the LV [1]. Occurring in about 30% of the patients with STEMI treated by primary PCI, LVR is a precursor for the development of heart failure (HF) and can also lead to arrhythmias and other complications, increasing cardiovascular morbidity and mortality [1]. Therefore, the identification of patients with a high likelihood of LVR has essential implications for risk stratification after AMI. Early detection and prompt therapeutic measures are crucial in order to improve the quality of life and survival in this high-risk group.

Prior data advocates that inflammation plays a critical role in the initiation and progression of atherosclerosis, but also in the resolution and healing that occurs after AMI [2]. The onset of myocardial ischemia triggers an initial pro-inflammatory response that promotes cardiac repair by mobilizing fibroblasts into the interstitial space and facilitating angiogenesis [3,4]. Then, an anti-inflammatory reparative phase follows, with the purpose of wound healing and scar formation, preventing complications such as cardiac rupture [3]. Persistent inflammation in the infarcted myocardium can exacerbate acute myocardial ischemia-reperfusion injury, favouring adverse LVR or ventricular aneurysm formation [3,4,5]. Cytokines are an important subset of inflammatory markers released in response to acute ischemia to modulate tissue repair and adaptation after injury [6]. Their elevated levels have been associated with adverse remodelling and adverse outcomes after AMI [7,8]. In this study, we will focus our attention on three inflammatory cytokines: IL-6, IL-1RA and resistin.

### 1.1. Interleukin 6 (IL-6)

IL-6 is an inflammatory cytokine involved in vascular inflammation, the initiation and progression of atherosclerosis and degradation of fibrous cap contributing to plaque instability [9]. It propagates inflammation in patients with AMI, and its levels at admission are associated with infarct size and cardiac function, making it a predictor of in-hospital prognosis [10], but also of LVR and long term outcome/mortality [9,11]. IL-6 levels are increased during the first 2 weeks and reach a steady-state afterward [12]. On the one hand, IL-6 has protective effects—such as myocytes protection against oxidative stress. On the other hand, it has been shown that IL-6 signalling can lead to hypertrophy and depressed cardiac function [13,14].

### 1.2. Interleukin 1 Receptor Antagonist (IL-1RA)

Interleukin-1 receptor antagonist is a competitive inhibitor of the pro-inflammatory cytokine IL-1, released as an acute phase reactant, modulating the inflammatory response [15]. IL-1RA production is induced by IL-1β and, thus, acts as a counter-regulator of IL-1β mediated processes [16]. It is involved in coronary atherosclerosis, ischemia-reperfusion injury (ischemia triggers IL-1RA synthesis in cardiomyocytes) and tissue inflammation [16]. In contrast to IL-1, for which a direct measurement is not applicable due to extremely low plasma levels, the level of circulating IL-1RA can be reliably quantified [17], therefore serving as a detectable surrogate marker for high IL-1β activity [17,18]. IL-1RA levels peaked right after PCI, decreased markedly after day 1 and then remained elevated even one year after STEMI [19]. IL-1RA levels correlate with the severity of inflammation [15], proving to be both a sensitive diagnostic [20] and prognostic marker in patients with acute coronary syndrome (ACS) [21].

### 1.3. Resistin

Resistin is a pro-inflammatory adipocytokine secreted predominantly by macrophages and adipocytes, with an important role in the pathogenesis and development of atherosclerosis (induces endothelial dysfunction, arterial inflammatory response and lipid accumulation in foam cells) [22]. It also upregulates the expression of other pro-inflammatory cytokines, including TNF-α, IL-6, IL-1β and monocyte chemoattractant protein-1 resistin in an NF-KB signalling dependent mechanism, thus promoting the inflammatory process [23]. Resistin levels are high in patients with ACS, its levels increasing early at 3–6 h after onset making it a potentially useful diagnostic marker [24]. Studies on animal models have indicated that resistin may directly affect the myocardium, promoting cardiac hypertrophy and dysfunction [25,26,27]. This way, resistin participates in obesity-related diseases, such as dyslipidaemia, atherosclerosis and cardiovascular disease, a fact also proved in human studies emphasizing its potential prognostic role [28].

Traditionally, studies have focussed on the association between single cytokines and LVR or MACE following AMI. However, there has been increasing recognition that inflammation following AMI is a complex process and assessing multiple cytokines may be beneficial [29,30,31,32].The aim of this study was to explore the potential role of a group of three inflammatory chemokines (IL-1RA, IL6, resistin) as an independent prognostic marker for LVR assessed by 3D echocardiography and adverse outcome in patients with STEMI.

## 2. Materials and Methods

### 2.1. Study Subjects

Patients with a first STEMI admitted to our hospital and treated by primary PCI (successfully reperfused) were prospectively enrolled in this study between 2019–2020. Patients with a previous myocardial infarction, severe comorbidities, significant renal/hepatic/respiratory failure (prior to the acute coronary event), recent stroke, recent surgery or trauma (under one month), active malignancy, acute myocarditis, other concomitant inflammatory disease (acute infections, autoimmune diseases), patients with addictions/poor compliance or those who refused to sign the informed consent were excluded.

Written consent was obtained from all patients who agreed to participate in this trial in accordance with the Declaration of Helsinki. This study was approved by the Ethics Committee from our hospital.

### 2.2. Blood Sample Collection and Storage

Whole blood samples (2 mL) were obtained by peripheral venous puncture between 24 and 48 h after admission for STEMI and at 6 months follow up. Blood was harvested on EDTA tubes and centrifuged (1000× *g*) for 15 min at −4 °C within 30 min of collection. After centrifugation, plasma (the supernatant) was aliquoted in Eppendorf tubes (300 µL each) and immediately frozen at −80 °C.

### 2.3. Pro-Inflammatory Cytokines Assay

For quantification of cytokine plasma levels at baseline and at 6 months follow up, we used the enzyme-linked immunosorbent assay (ELISA) method. IL-1RA, Il-6 and resistin were assayed using R&D Systems kits designated for each cytokine and following the corresponding protocol (Quantikine ELISA Human IL-1ra/IL-1F3 Immunoassay, Quantikine ELISA Human Il-6 Immunoassay, Quantikine ELISA Human Resistin Immunoassay, R&D Systems Inc., Minneapolis, MN, USA). The results were expressed in terms of absorbance using a microplate reader set to 450 nm, and concentrations were obtained from the standard curve using the provided formula. The mean normal value for IL-1RA was 309 pg/mL with a limit of detection of 2.2–18.3 pg/mL (mean value 6.3 pg/mL); the coefficient of variance for intra-assay precision was 5–7.3% and for inter-assay precision 8–11%. The normal range mean for IL-6 was 3.13 pg/mL, with a limit of detection of 0.70 pg/mL; the coefficient of variance for intra-assay precision was 1.6–4.2% and for inter-assay precision 3.3–6.4%. The normal values for resistin were between 5 and 24.5 ng/mL, with a mean of 11.9 ng/mL and a limit of detection of 0.010–0.055 ng/mL (mean value 0.026 pg/mL); the coefficient of variance for intra-assay precision was 3.8–5.3% and for inter-assay precision 78–8.2%. No significant cross-reactivity or interference was encountered.

All samples were assayed in the Laboratory of molecular cardiology, cardiology department, Clinical Emergency Hospital Bucharest.

### 2.4. Echocardiography

Standard 2D transthoracic echocardiography was performed for all patients using the GE VIVID E9 ultrasound system. In addition to conventional parameters, we also measured LV global longitudinal strain (LV GLS) and LV mechanical dispersion using 2D speckle tracking imaging. For a better morphological and functional analysis of the left ventricle, 3D echocardiography was performed: measurement of LV end-systolic volume (LVESV), LV end-diastolic volume (LVEDV), 3D LVEF.

All measurements were performed at baseline (between 3 and 5 days after STEMI) and at 6 months follow up. Stored data was analyzed offline on EchoPAC work stations. Image acquisition and measurements were performed according to the recommendation/guidelines of the European Association of Cardiovascular Imaging [33] and the American Society of Echocardiography [34]. The measurements were performed by two independent operators trained in cardiac ultrasound.

### 2.5. Coronary Angiography

All patients included in this study underwent coronary angiography at admission according to STEMI treatment guidelines [35].

Follow up and outcomes.

All patients were followed up at 6 months (clinical examination, blood sample collection, standard echocardiography, strain analysis, 3D echocardiography).

There were two primary endpoints in this study:

(1) LV remodelling—defined as an increase of LVEDV by more than 20% at 6-month follow-up compared with baseline values;
(2) MACE—defined as death, hospitalization for recurrent ischemia/reinfarction or hospitalization for HF that occurred during the 6 months of follow up. For accurate results, we asked our subjects to provide the hospital discharge papers at the 6 months follow up if hospital admission (in case of MACE) was required during the follow-up period.

### 2.6. Statistical Analysis

Statistical analysis was performed using SPSS software (IBM SPSS Statistics v.22.0). Categorical data were presented as frequencies and percentages while normally distributed, continuous variables were reported as mean ± SD. Statistical comparisons were performed with χ2 and Fisher’s tests for categorical variables and with Student’s *t*-tests for continuous variables. The KolmogorovSmirnov test confirmed the normal distribution of the continuous variable.

To determine the predictors of adverse LVR and MACE, all demographic characteristics, laboratory measurements and procedural factors were evaluated using binary logistic regression analysis. Variables with statistical significance in univariable analysis were further incorporated in a multivariate analysis providing odds ratio (OR) and 95% confidence intervals (CI). COX multivariate regression analysis was used to determine significant predictors for MACE.

A receiver operating curve (ROC) analysis was conducted in order to calculate the area under the curve (AUC) to assess the ability of the tested parameters to predict LVR and adverse outcome/MACE. The AUC results were considered excellent for AUC between 0.9–1, very good for AUC 0.8–0.9, good for AUC between 0.7–0.8, satisfactory between 0.6–0.7 and unsatisfactory between 0.5–0.6. Cut-off values were determined based on the Youden index. *p* values < 0.05 were considered statistically significant.

## 3. Results

### 3.1. Baseline Characteristics

Out of 53 patients initially included in this study, 7 were lost at follow up and 5 had poor acoustic window leaving a final study population of 41 patients with STEMI treated by primary PCI (mean age was 49.1 ± 9.34 years, 82.2% men). Out of 41 patients, 2 died, and 8 (20%) showed LV remodelling at 6 months follow up (assessed by 3D transthoracic echocardiography).

Thus 41 patients were included in the final analysis. The mean age was 49.1 ± 9.34 years, 82.2% were males, 46.3% were hypertensives, 17.1% diabetics, 85.4% were smokers and 75.6% had dyslipidaemias.

Baseline demographic and clinical characteristics for the entire cohort, according to the presence of adverse LVR, are presented in Table 1. At discharge, all patients received standard medical therapy in accordance with current clinical practice guidelines [35].

### 3.2. Echocardiographic Parameters

Echocardiography was performed at baseline (T0) and at 6 months follow up (T6). The study population was dichotomized according to remodelling status. General characteristics of patients with or without remodelling are depicted in Table 1.

LVEF differed significantly between the LV remodelling and non-remodelling groups (*p* = 0.001). The mean 3D LVEF at follow up was increased compared to the baseline (40.02 ± 8.05 vs. 46.74 ± 8.34, *p* < 0.001). LV GLS increased at follow up from −12.44 ± 4.17 to −14.78 ± 4.19, *p* < 0.001. LV mechanical dispersion did not differ significantly between the baseline and 6 months follow up (65.94 ± 24.4 vs. 62.53 ± 20.9, *p* = 0.340). Echocardiographic parameters at baseline and at 6 months follow up are reported in Table 2 and Table S1.

During follow up the administration of dual antiplatelet therapy, beta-blockers, angiotensin converter enzyme inhibitors and statins were also not significant between groups.

### 3.3. Left Ventricular Remodelling

At 6 months follow up, the incidence of adverse LVR was 20.5%. Baseline characteristics and echocardiographic parameters for patients with or without LVR are summarized in Table 1 and Table 2.

The incidence of risk factors associated with cardiovascular disease did not differ significantly between the two groups. In comparison to the non-remodelling group, patients with adverse LVR had higher peak CK-MB levels, higher white cell blood count, higher glycaemic values and lower haemoglobin levels, as seen in Table 1. The LVR group had a higher rate of LAD stenosis as culprit lesion compared to the no-remodeling group (71.4% vs. 43.8%, *p* = 0.55).

It is worth mentioning that no residual ischemia was detected at the follow-up.

The link between LVR and the other parameters was examined. By using binary univariate regression analysis, the occurrence of adverse LVR at 6 months follow up was significantly associated with Killip class (*p* = 0.007), 2D LVEF (*p* = 0.005), 3D LVEF (*p* = 0.009) GLS LV (*p* = 0.005), LV mechanical dispersion (*p* = 0.005), E/e (*p* = 0.002), glycemia (*p* = 0.02), haemoglobin levels (*p* = 0.025) and peak CK-MB levels (*p* = 0.009), as seen in Table 3. Univariate binary logistic regression analysis identified variables correlated with LVR. After checking for collinearity GLS, mechanical dispersion, E/e’ and CK-MB were further analyzed by multivariate binary regression form which only CK-MB remained a predictor of LVR.

### 3.4. Cytokines Expression and LVR

Circulating plasma levels of Il-1RA, IL-6 and resistin were measured to test the potential role of these inflammatory markers in post-AMI adverse LVR. In the entire cohort, Il-1RA, Il-6 and resistin plasma values decreased from the baseline at 6 months follow up, as seen in Table S2.

**Figure 1:**
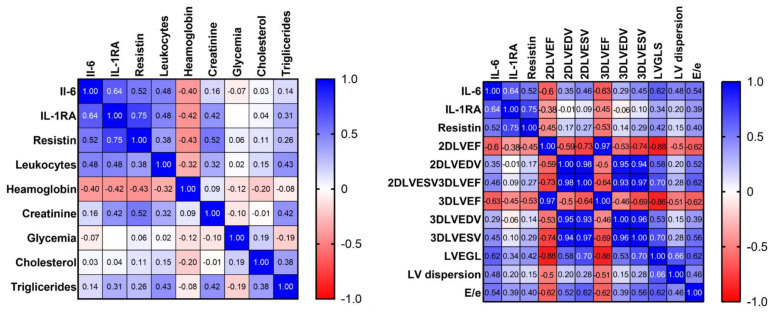
Correlation matrix: correlations between cytokines and biochemical and echocardiographic parameters.

Correlations between cytokine values at baseline and echocardiographic and biochemical parameters are depicted in the heatmaps below Figure 1.

**Figure 2:**
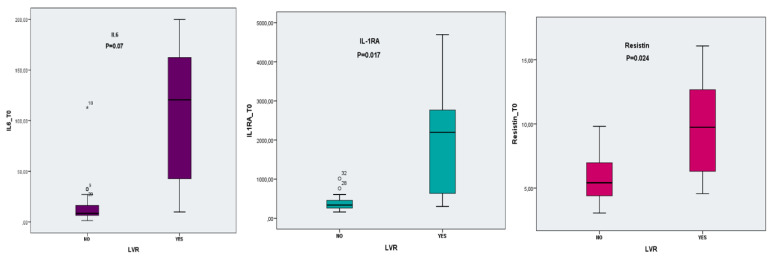
Cytokine levels were significantly higher in the remodelling group.

The plasmatic values of cytokines at baseline differed significantly between the LVR and non LVR group as follows: IL-6 (107.53 ± 69.11 vs. 14.91 ± 20.21, *p* = 0.07), Il-1RA (2024.23 ± 1476.57 vs. 394.76 ± 178.11, *p* = 0.017) and resistin (9.76 ± 3.96 vs. 5.73 ± 1.86, *p* = 0.024), as seen in Figure 2. No significant difference was found at 6 months follow up.

Univariate binary logistic regression analysis identified each of the three cytokines as a predictor for adverse LVR at 6 months follow up (*p* < 0.05), as seen in Table 4. These variables were included in a multivariate analysis model: the logistic regression model was statistically significant, Chi-square = 27.657, *p* = 0.000; it explained 80.4% of the variance in the LVR (Nagelkerke R square = 0.804) and correctly classified 97.4% of cases; IL-1RA and IL-6 was proven to have a greater contribution to the model than resistin.

All three cytokines had good prediction abilities for LVR with AUC greater than 0.7: AUC 0.940 (95% CI: 0.838–1), *p* = 0.000 for IL-6; AUC 0.859 (95% CI: 0.680–1), *p* = 0.002 for IL-1RA; AUC 0.825 (95% CI: 0.641–1), *p* = 0.005 for resistin. For each variable we determined a cut-off value, based on the maximum value of the Youden index as follows: 806.79 pg/dL (Se = 75%, Sp = 96.8%) for IL-1Ra, 34.8 pg/dL (Se 87.5%, Sp 96.8%) for Il-6 and 6.9 ng/dL (Se 75%, Sp 80%) for resistin. Out of the three variables, IL-6 proved to have the best predictive value for LVR, as shown in Table 5.

We further analyzed the patients based on the number of biomarkers with values above the cut-off and divided them into four groups—with one, two or three biomarkers over the cut-off or none. We found a significant difference between LVR and no LVR groups regarding the number of markers over the cut-off (*p* = 0.000). Out of the patients from the LVR group, 71.4% had three biomarkers over the cut-off, 14.3% had two biomarkers and 14.3% had only one biomarker over the cut-off. In the non-LVR group, 71.9% had no marker over the cut-off, 25% had one marker over the cut-off and only 3.1% had two markers over the cut-off. None of the patients from the non-LVR group had three markers over the cut-off value.

**Figure 3:**
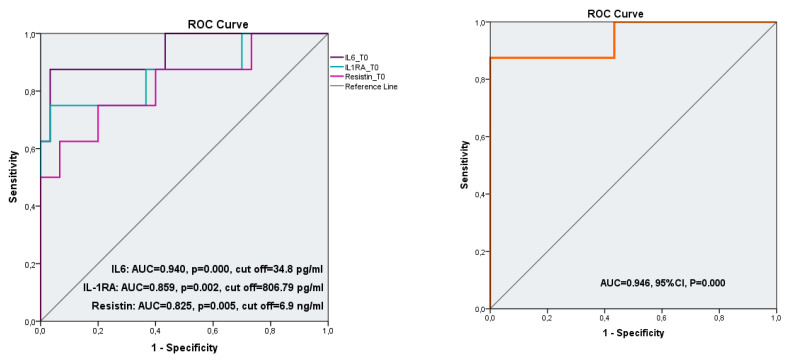
ROC curves of univariate variables (Il-6, IL-1RA, resistin) at admission for predicting LV (**left**); The ROC curve for risk prediction model (simultaneously including the three cytokines) (**right**).

The three cytokines as a group showed a higher predictive performance for LVR than each separate variable in the ROC model simultaneously, including the three cytokines that yielded AUC of 0.946, CI 95% and *p* = 0.000, as shown in Figure 3.

In addition, the cytokines as a group had better predictive value than the other clinical and echocardiographic parameters that proved to be predictors of LVR in univariate analysis, thus proving their predictive power, as shown in Figure S1.

### 3.5. Clinical End Points—MACE

Patients were classified into the MACE group (patients who experienced any of the MACE in the first 6 months after STEMI) and patients without MACE. During the follow-up period, 22% of patients reached the secondary endpoint: 4.8% cardiac deaths, 7.31% readmissions for angina, 12.19% readmissions for heart failure exacerbation.

ROC statistical analysis showed that 3D LVEF < 36% (Se 77.8%, Sp 78.1%), LV GLS < −11 (Se 87.5%, Sp 84.4%), LV mechanical dispersion > 65.5 (Se 88.9%, Sp 81.2%), E/e’ > 10.3 (Se 75%, Sp 83%), peak CK-MB > 293 U/L (Se 75%, Sp 78.1%) were the best cut-off values for predicting MACE during the 6 months of follow up in patients with STEMI treated by primary PCI.

Univariate binary regression analysis demonstrated a significant association between MACE and the three cytokines: OR = 1.027, *p* = 0.005 for IL-6; OR = 1.002, *p* = 0.013 for IL-1RA and OR = 1.704, *p* = 0.004 for resistin. Introducing the variables in a multivariable COX regression model, all three remained independently associated with MACE (Chi-square = 20.289, *p* model = 0.000)—Table 6.

The three cytokines had good prediction abilities for MACE, with AUC greater than 0.7: AUC 0.852 (95% CI), *p* = 0.001 for IL-6; AUC 0.814 (95% CI), *p* = 0.004 for IL-1RA; AUC 0.842 (95% CI), *p* = 0.002 for resistin, as shown in Figure S1. For each variable we determined a cut-off value, based on the maximum value of the Youden index as follows: 456.9 pg/mL (Se = 77.8%, Sp = 75%) for IL-1RA, 25.5 pg/dL (Se 87.7%, Sp 81.2%) for Il-6 and 6.9 ng/dL (Se 77.8%, Sp 80.6%) for resistin, as shown in Table 7. Out of the three variables, IL-6 proved to have the best predictive value for LV remodelling. IL-6, IL-1RA and resistin as a group had the best predictive value with AUC = 0.875, *p* = 0.000, as shown in Figure S2.

We conducted a further analysis based on the number of cytokines with values above the cut-off (determined above) and divided the patients into four groups (with one, two or three cytokine values above the cut-off or none above the cut-off). There was a significant difference regarding the number of markers over cut-off between MACE and no MACE groups (*p* = 0.000). Our results showed that in the MACE group, 77.8% of patients had three markers with values above the cut-off, 11.1% had two and 11.1% had only one cytokine over the cut-off. In the group without MACE, no patient had three markers over cut-off, 12.5% had two markers over the cut-off, 18.8% had one and the majority (68.8%) had no markers over the cut-off value.

**Figure 4:**
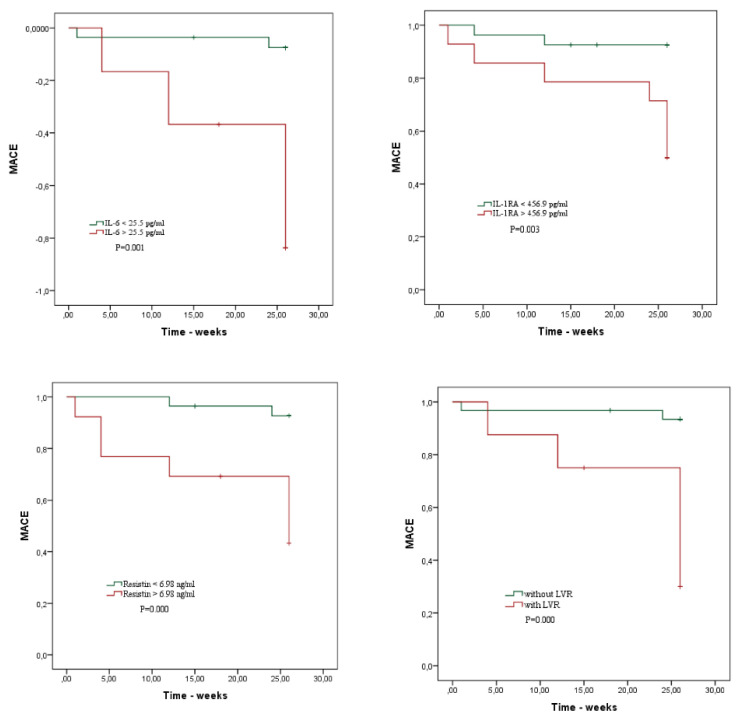
Kaplan-Meyer analysis-Kaplan-Meier curves showing the risk of MACE stratified by IL-6, IL-1RA, resistin and LVR. IL-6, IL-1RA and resistin were dichotomized according to the optimal cut-off value, calculated by ROC analysis.

Survival curves showing the risk of MACE in relation to IL-6 (log Rank Chi-square 11.589, *p* = 0.001), IL1-RA (log-rank Chi-square = 9.108, *p* = 0.003) and resistin (log-rank Chi-square 12.418, *p* = 0.000) are depicted below in Figure 4. Patients with cytokine values higher than the cut-off seem to have a higher incidence of MACE, as well as patients with LVR at follow up as expected (log-rank Chi-square = 14.841, *p* = 0.000).

## 4. Discussion

In our cohort of STEMI patients treated by primary PCI we found that plasmatic cytokine levels measured within 48 h from symptom onset are independent predictors of adverse LVR at 6 months follow-up. They are also associated with worse LV function and worse patient outcomes, high cytokine levels being correlated with a higher incidence of MACE/adverse events. Our findings indicate that IL-6, IL1-RA and resistin have a promising role as prognostic biomarkers in STEMI (for risk stratification, potentially guidance of clinical care and as therapeutic targets).

Emerging evidence suggests that cytokine levels are important predictors of cardiac events and mortality in multiple cardiac diseases. Cardiac remodelling is one of the major determinants of HF development after AMI and has been associated with worse outcomes. It occurs as a consequence of an increase in wall stress determined by distension of the infarcted area and cardiomyocyte loss [36,37]. There is clinical and experimental evidence showing that systemic and local inflammation is associated with LVR [38]. In the setting of acute myocardial ischaemia, the complex inflammatory response accompanied by a release of several cytokines is essential for proper infarct healing and scar formation [39,40]. However, as previously mentioned, an exaggerated and persisting inflammatory response leads to an increase in myocardial tissue damage and subsequently worse clinical outcomes [3].

### 4.1. IL-6

It is well known that IL-6 concentrations are significantly higher in STEMI patients compared to healthy controls [41]. Lately, an association between circulating IL-6 levels and the extent of myocardial necrosis has been described in a large cohort of 1028 STEMI patients [42]. In our investigation, the concentration of IL-6 > 34.8 pg/dL (AUC 0.940, *p* = 0.000) was an independent predictor of LVR. IL-6 plasmatic levels at admission were higher in patients with LVR (107.53 ± 69.11 vs. 14.91 ± 20.21, *p* = 0.07) and in patients with MACE (88.91 ± 74.48 vs. 18.90 ± 26.6, *p* = 0.023). Il-6 levels decreased at 6 months follow up from 34.27 ± 5 pg/mL at baseline to 5.6 ± 7.8 pg/mL. This corresponds to recently published data also showing that higher levels of circulating plasma IL 6-in patients with STEMI are associated with larger infarct size, worse myocardial function, higher likelihood of LVR and decreased cardiac function at 4 months [12,40]. Groot et al. measured IL-6 levels at hospital admission, 24 h, 2 weeks, 7 weeks, 4 months and 1 year follow up [12]. At 24 h after admission, IL-6 had increased threefold compared to baseline (*p* < 0.001) and returned below baseline (*p* < 0.001) 2 weeks after STEMI [12]. IL-6 at 24 h was independently associated with infarct sizeβ 5.4 (95% CI 3.3–7.5); *p* < 0.001 and higher levels were associated with lower LVEF [12]. Tiller et al. measured IL-6 levels at 24 h after admission (similar to our research). High concentrations of IL-6 > 17 ng/l were independently associated with worse myocardial function, larger infarct extent, more severe reperfusion injury, and a higher likelihood for LVR, suggesting IL-6 as a useful biomarker of more serious outcome and potential therapeutic target [40]. Other trials also found a significant association between IL-6 and MACE either by univariate or multivariate analysis [30,43,44].

Its important role in LVR was best emphasized by two trials. First, Zhao et al. demonstrated in a study on mice that genetic deletion of IL-6 ameliorates LVR remodelling and systolic dysfunction after myocardial infarction, confirming the harmful role of IL-6 in LVR after acute myocardial ischemia/reperfusion injury. Modulation of IL-6 signalling may therefore have therapeutic potential for patients after MI at risk for adverse remodelling and development of the heart [45]. Second, in 2021, a recombinant humanized monoclonal antibody that binds to the IL-6 receptor (Tocilizumab) to block its signal transmission was tested on humans, having a beneficial effect by increasing myocardial salvage in a group of 199 patients with acute STEMI—a significant difference in infarct size was assessed by CMR (7.2% vs. 9.1% of myocardial volume, *p* = 0.08) [46].

### 4.2. IL-1RA

In our study, IL-1RA levels were higher in patients with LVR at follow up compared to those without (2024.23 ± 1476.57 pg/mL vs. 394.76 ± 178.11 pg/mL, *p* = 0.017); higher IL-1RA levels were also observed in patients with MACE within 6 months (1643.703 ± 1544 pg/mL with MACE vs. 456.222 ± 371.75 pg/mL without MACE, *p* = 0.05). Their levels decreased over time from 589.65 ± 856.31 pg/mL at baseline to 447.98 ± 184.91 pg/mL at 6 months, demonstrating that it is an acute phase reactant. At 6 month follow up there was no significant difference between groups (LVR/without LVR, MACE/without MACE).

IL-1RA is an inflammatory chemokine that blocks the binding of IL-1; it is a more reliable marker of IL-1 system activation and easier to detect than Il-1 β. Considering this, many studies used Il-1RA as a detectable surrogate parameter for IL-1β activity. Consistent with our findings, previous trials demonstrated an increase of IL-1RA levels in the acute phase of AMI and also an association between IL-1RA and myocardial necrosis [10,11,15,47]. Patti et al. found that IL-1RA values were significantly higher in patients with AMI than in those with angina (671 vs. 320 pg/mL, *p* = 0.013) [15].

In our study, IL-1RA proved to have a prognostic value as an independent predictor of adverse LVR after STEMI (OR = 1.004, *p* = 0.05, Chi-square 19.380, Wald 3.71) and also a predictor of MACE (OR = 1.002, *p* = 0.013, Chi-square 10.919, Wald 6.15) in univariate binary logistic regression. The association was confirmed in multivariate logistic regression as well. Cut-off values of IL-1RA were obtained by ROC analysis for LVR (806.79 pg/mL, Se 75%, Sp 96.8%, AUC 0.859, *p* = 0.002) and MACE (456.9 pg/mL, Se 77.8%, Sp 80.6%, AUC 0.814, *p* = 0.004). Our results regarding the prognostic role of IL-1RA were consistent with previous research. A metanalysis from 2017 found a positive association between serum IL-1RA levels and the risk of cardiovascular disease in a case-cohort study on 1855 patients [18]. In 2018, Schofer et al. demonstrated that IL-1RA is an independent predictor of cardiovascular mortality, beyond the prognostic value of CRP and troponin T, in a group of patients with ACS and known CAD (HR 1.93, 95% CI 1.33–2.80, *p* < 0.001) [17]. Supporting its emerging role as a predictor of adverse outcome, the associated inflammatory cytokines TNF-α, IL-6 and IL-1Ra had significantly higher values in patients with MI complicated with cardiogenic shock than in those with a more favourable evolution. Between the three, Il-1RA seemed to be a promising diagnostic biomarker, predicting poor patient evolution [10]. Furthermore, the potential role of IL-1 in LVR prediction was debated by Hilde et al. who found an independent association between sIL-1R2 levels bunt not IL-1RA and CMR parameters of adverse LVR following STEMI [19].

Several studies show the benefits of intervening in the inflammatory cascade during/after MI. Targeting the IL-1β pathway with canakinumab led to a lower rate of recurrent cardiovascular events than placebo but with no significant difference in all-cause mortality in a group of patients with previous myocardial infarction and high CRP levels [48]. However, the results from CANTOS were unable to be implemented into clinical practice due to the significantly elevated risk of fatal infections among patients receiving canakinumab vs. placebo [48].

### 4.3. Resistin

We concluded that resistin levels were higher in patients with LVR (9.76 ± 3.96 ng/mL in LVR group vs. 5.73 ± 1.86 ng/mL in the group without LVR, *p* = 0.024) and in patients who experienced MACE during the follow up period (9.94 ± 3.76 ng/mL vs. 5.76 ± 1.94 ng/mL, *p* = 0.01). Resistin levels decreased after 6 months in all patients (5.6 ± 7.8 ng/mL at baseline vs. 5.39 ±2.68 ng/mL at follow up). In addition, it is worth mentioning that the difference between patients with or without LVR/MACE was lost at 6 months follow up (5.23 ± 2 ng/mL with LVR vs. 6.1 ± 2.1 ng/mL without LVR, *p* = 0.273 and 6.67 ± 2.9 ng/mL with MACE vs. 5.76 ± 1.95 ng/mL without MACE, *p* = 0.354).

Similar results were observed by Lubos et al., who proved that resistin might have a role as a diagnostic and prognostic marker considering its elevated levels in patients presenting with ACS (STEMI, NSTEMI, unstable angina) [22]. According to his research, resistin levels rose at 3–6 h after chest pain onset, peaking at over 12 h after chest pain onset (5.74 ng/mL) [22]. Increased levels of resistin >6.31 ng/mL were associated with a 1.22-fold (95% CI 1.04–1.43; *p* = 0.02) risk for future fatal cardiovascular events [22]. This is in concordance with the results obtained by Chu et al., who observed significantly elevated resistin levels within the first week after ACS onset, also found a positive correlation between resistin levels and myocardial injury markers and a negative correlation with LVEF [49]. This is probably explained by recent evidence showing that resistin is related to plaque instability [50]. In this trial from 2018, the levels of resistin were higher in the serum of ACS patients (more than twice as high) and also in vulnerable atherosclerotic plaques [50]. High concentration of resistin encourage fibrous atherosclerotic cap to become more rupture-prone by converting vascular smooth muscle cells proliferation into apoptosis, thus preventing fibrous cap repair [51]. Resistin is overexpressed during myocardial ischemia through the ERK/MAPK pathway [52].

Muse et al. demonstrated in a large cohort study the association between high resistin levels and the incidence of cardiovascular events (AMI, unstable angina, stroke, HF) [53]. Other studies also confirmed that the levels of resistin corelate with cardiac fibrosis and that resistin is an independent predictor of LVR in patients with STEMI and metabolic syndrome at 12 months follow up [54]. Erer et al. emphasised the role of resistin as an independent predictor for MACE in patients with STEMI (OR = 1.11, 95% CI, *p* = 0.03) [55]. All these findings are consistent with the results from our study that proved the role of resistin as an independent predictor of LVR (OR = 1.7, *p* = 0.007, Chi-square = 11.813, Wald index = 7.26) with best cut-off value of obtained by ROC analysis of 6.9 ng/mL, Se = 75%, Sp = 80% (AUC 0.825, *p* = 0.005) and also an independent predictor of MACE (OR = 1.7, *p* = 0.004, Chi-square = 13.551,Wald index = 8.39), with a cut-off value of 6.98 ng/mL, Se 77.8%, Sp = 80.6% (AUC 0.842, *p* = 0.002). Interestingly, in opposition with the studies mentioned above and with the results from our research, Gao et al. demonstrated in a preclinical trial the cardioprotective effect of resistin, with reduction of apoptosis and infarct size and protection against I/R injury, when administered prior to ischemia induction in a mouse model [56].

### 4.4. Cytokines as a Group

Considering that inflammation following STEMI is a complex process, investigating multiple cytokines may be beneficial in predicting patient evolution more accurately.

As a basis for the inclusion of these particular three cytokines in the current study, each of them has been associated with adverse outcomes following ACS in at least one prior study (as detailed above). There were a few trials that assessed a combination of various markers as predictors in ACS. Novo et al. developed a risk score from the analysis of 27 inflammatory cytokines to predict outcome in patients with AMI; they concluded that the presence of more than 13 cytokines above the median level was a better predictor of MACE with an AUC 0.720 [30]. The combination of GDF-15 and TRAIL-R2 was a better predictor of long-term all-cause mortality in patients with acute myocardial infarction in a study from 2017 [44]. Kilic et al. studied the relation between pro-inflammatory to anti-inflammatory cytokine ratios and long-term prognosis in patients with NSTEMI [31]. They analyzed the serum concentrations of CRP, IL-1β, IL-6 and TNFα, IL-10 and calculated pro-inflammatory to anti-inflammatory cytokine ratios (each cytokine divided by IL-10) [31]. Logistic regression analysis found IL-6/IL-10 ratio as the most important predictor for new coronary event (OR = 2.24, *p* = 0.006) [31]. Chalikias et al. used the same principle of ratios between pro- and anti-inflammatory markers and proposed the IL-18/IL-10 ratio (association between the pro-inflammatory cytokine IL-18 and the anti-inflammatory cytokine IL-10) as a predictor of recurrent coronary events during long-term follow-up in patients presenting with ACS (OR 1.91, 95% CI 1.37–2.65, *p* < 0.001) [29]. In a more recent trial from 2020, Kristono et al. proved that the IL-6-IL-8 score is an independent predictor of MACE at one year follow up after STEMI and a stronger predictor than any individual cytokine (OR = 2.77, *p* = 0.007) [43].

To our knowledge, so far, no study has looked into combining Il-1RA, IL-6 and resistin as predictors for LVR and MACE after STEMI. This research concluded that the combination of IL-6, IL1-RA and resistin is a better prognostic marker for both MACE and LVR than each cytokine taken separately (as illustrated in Table 5 and Table 7, respectively), and also compared to some echocardiographic or biological parameters assessed separately. There is also the possibility that the higher level of cytokines is the result of the impaired LV function detected at baseline rather than being only determinants of LVR and MACE at follow up. Even though further studies are required to better understand the exact role of inflammation in acute coronary events, our findings hold great potential for the prognostication of LVR and MACE, treatment monitoring and why treatment should be tailored according to risk stratification and potential treatment target in patients with STEMI. In support of the latter come recent trials targeting the IL-1β pathway with Canakinumab and IL-6 with Tocilizumab, both with promising results in patients with ischemic heart disease [46,48].

There is more and more evidence of the importance of inflammatory cytokines in cardiovascular diseases and considering all the research supporting this, they should be added to the list of biomarkers providing prognostic information and potentially guiding clinical care in order to improve the outcome of AMI patients [11].

### 4.5. Limitations

Our study had several limitations. First, the lack of a separate validation cohort. Second, it is worth mentioning that we explored the role of inflammation in “mid term LVR” at 6 months; we do not know what happens after a longer period of time. Third, considering the many participants in the inflammatory cascade, other inflammatory cytokines used in previous studies could have been of potential use in this research but were not able to be included in this study due to limited financial resources. Fourth, considering the small sample population, the performance and precision of predictions may have been affected; therefore, larger further studies are required to validate our findings. In addition to the small sample size, there are missing data that can interfere with the interpretation of the results. Inflammatory-linked determinants of the LVR were not considered in the analysis, such as the presence of coronary microvascular dysfunction [57] and the achievement of satisfactory control of LDL-cholesterol [58].

## 5. Conclusions

IL-6, IL1-RA and resistin have a promising role as prognostic biomarkers both as independent markers and also as a group in patients with STEMI. They hold potential for future use in clinical practice for risk stratification, guidance of clinical care and as therapeutic targets.

## Figures and Tables

**Table 1:** Baseline characteristics of the entire study population and divided into two subgroups according to the occurrence of LVR at follow up.

	Study Population (*n* = 41)	LVR (*n* = 9)	Without LVR (*n* = 30)	*p* Value
Clinical characteristics				
Age (years)	49.1 ± 9.34	49.38 ± 11.5	49.06 ± 9.14	0.936
Cardiovascular risk factors				
Smoking	85.4%	20.6%	79.4%	0.976
Obesity	22%	22.2%	77.8%	0.885
Hypertension	46.3%	11.8%	88.2%	0.234
Dyslipidaemia	75.6%	17.2%	82.8%	0.389
Diabetes	17.1%	33.3%	66.7%	0.398
Metabolic syndrome	12.2%	40%	17.6%	0.248
Clinical presentation				
Killip class ≥ 2	17%	100%	0%	0.000
Angiographic characteristics				
LAD	51.2%	71.4%	43.8%	0.55
RCA	39%	28.6%	43.8%	
LCX	7.3%	0%	12.4%	
Multivessel CAD	36.6%	13.3%	86.7%	0.380
Occluded artery	58.5%	26.1%	73.9%	0.301
PCI over 12 h	15%	37.5%	62.5%	0.182
Laboratory characteristics				
WBC count, × 10^3^/mm^3^	11,260 ± 3628	15,762.85 ± 3674.59	9710.93 ± 2092.57	0.002
Haemoglobin, g/dL	14.06 ± 1.44	13 ± 0.97	14.49 ± 1.44	0.014
Creatinine (mg/dL)	0.83 ± 0.23	0.89 ± 0.38	0.8 ± 0.15	0.55
Glycemia (mg/dL)	118.02 ± 38.62	153.50 ± 53.58	107.66 ± 28.9	0.002
Cholesterol (mg/dL)	217.21 ± 64.36	211.00 ± 72.29	219.04 ± 65.26	0.76
Triglycerides (mg/dL)	202.37 ± 181.288	236.50 ± 300.255	199.04 ± 143.85	0.62
Peak CK-MB (U/L)	251.58 ± 211.26	403.75 ± 183.77	182.77 ± 119.011	0.000
Follow up				
MACE	22%	28.6%	71.4%	0.002
Ventricular arrhythmias	7.3%	100%	0%	0.046
Atrial fibrillation	7.3%	33.3%	66.7%	0.101

**Table 2:** Echocardiographic parameters at baseline for the entire study population and divided into subgroups according to the occurrence of LVR at follow up.

	Population	LVR (*n* = 9)	Without LVR (*n* = 31)	*p* Value
2D LVEDV (ml)	107.29 ± 38.9	116.0 ± 32.43	103.32 ± 41.24	0.426
2D LVESV (ml)	66.41 ± 35.45	82.25 ± 27.05	60.48 ± 36.53	0.125
2D LVEF (%)	39.85 ± 8.9	29.62 ± 4.13	43.16 ± 7.03	0.000
3D LVEDV (ml)	114.63 ± 33.37	120.00 ± 31.27	112.22 ± 34.49	0.571
3D LVESV (ml)	70.09 ± 28.34	84.37 ± 25.47	64.87 ± 27.97	0.082
3D LVEF (%)	40.02 ± 8.05	30.37 ± 3.88	43.25 ± 5.97	0.000
LV GLS	−12.44 ± 4.17	−8.02 ± 1.83	−14.01 ± 3.4	0.000
LV mechanical dispersion	65.94 ± 24.4	101.57 ± 20.77	53.77 ± 10.21	0.000
E/e’ (LV filling pressure)	9.05 ± 3.04	12.61 ± 1.7	8.34 ± 2.44	0.000

**Table 3:** Univariate binary logistic regression to assess the ability of various parameters to predict LVR.

	Parameters	OR	*p*-Value
Clinical characteristics	Age	1.006	0.888
	KILLIP class	31.011	0.007
Parameters of LV function	2D LVEF	0.752	0.005
	GLS LV	1.791	0.005
	LV mechanical dispersion	1.068	0.005
	3D LVEF	0.615	0.009
	E/e’ ratio	2.17	0.002
Biological parameters	CK-MB max	1.012	0.009
	Leukocytes	1.002	0.030
	Haemoglobin	0.490	0.025
	Glycemia	1.028	0.020

**Table 4:** Univariate binary logistic regression for cytokines to assess the ability to predict LVR.

	Chi-Square	Wald	OR	*p*-Value
IL-6	19.005	8.094	1.042	0.004
IL-1RA	20.199	3.7	1.004	0.05
Resistin	11.813	7.26	1.7	0.007

**Table 5:** ROC analysis -performance of LVR prediction using plasma cytokines.

	AUC	Cut-off Value	Sensitivity (%)	Specificity (%)	*p*-Value
IL-6 (pg/mL)	0.940	34.8	87.5	96.8	0.000
IL1-RA (pg/mL)	0.859	806.79	75	96.8	0.002
Resistin (ng/mL)	0.825	6.9	75	80	0.005
Combination of cytokines	0.946				0.000

**Table 6:** Univariate and COX multivariate regression analysis for MACE prediction.

	Univariate Regression Analysis				COX Multivariate Regression Analysis		
	Chi-Square	Wald	OR	*p*-Value	Wald	*p*-Value	Chi-Square for Model
IL-6	12.142	7.87	1.027	0.005	7.304	0.007	20.289 *p* value 0.000
IL-1RA	10.919	6.152	1.002	0.013	3.985	0.046	
Resistin	13.551	8.39	1.704	0.004	5.366	0.021	

**Table 7:** ROC analysis—performance of MACE prediction using plasma cytokines.

Cytokines	AUC	Cut-off Value	Sensitivity (%)	Specificity (%)	*p*-Value
IL-6 pg/ml	0.852	25.5	87.7	81.2	0.001
IL1-RA pg/ml	0.814	456.9	77.8	75%	0.004
Resistin ng/ml	0.842	6.98	77.8	80.6%	0.002
Predicted probability of the combination of cytokines	0.875				0.000

## Data Availability

Data available upon request.

## Supplementary Materials

The following supporting information can be downloaded at: https://www.mdpi.com/article/10.3390/diagnostics12020266/s1, Table S1: Evolution of echocardiographic parameters in time (baseline vs 6 months follow) for the entire study population; Table S2: Evolution of inflammatory markers in STEMI (baseline vs 6 months follow up). Figure S1: ROC curves for CK-MB, LV-GLS, LV dispersion and E/e’ at admission for predicting LVR; Figure S2: ROC curves of univariate variables (Il-6, IL-1RA, resistin) at admission for predicting MACE; The ROC curve for risk prediction model (simultaneously including the three cytokines) (bottom right).

## Abbreviations

ACS	acute coronary syndrome
AMI	acute myocardial infarction
AUC	area under the curve
CAD	coronary artery disease
CI	confidence interval
CK-MB	Creatine kinase-MB
CMR	cardiac magnetic resonance
CRP	C reactive protein
EDTA	Ethylenediaminetetraacetic acid
ELISA	enzyme-linked immunosorbent assays
ERK/MAPK pathway	Extracellular signal-regulated kinase/mitogen-activated protein kinase
GDF-15	Growth/Differentiation Factor 15
GLS	global longitudinal strain
HF	heart failure
IL-10	Interleukin 10
IL-1RA	Interleukin 1 receptor antagonist
IL-1β	interleukin 1β
IL-6	interleukin 6
LAD	left anterior descending artery
LCX	left circumflex artery
LV	left ventricle
LVEDV	left ventricular end-diastolic volume
LVEF	left ventricular ejection fraction
LVESV	left ventricular end-systolic volume
LVR	left ventricular remodelling
MACE	major adverse cardiac events
NF-KB signalling	Nuclear Factor kappa B signalling
NSTEMI	non-ST elevation myocardial infarction
OR	odds ratio
PCI	percutaneous coronary intervention
RCA	right coronary artery
ROC	Receiver operating characteristic
Sen	Sensitivity
Sp	Specificity
STEMI	ST elevation myocardial infarction
TNFα	Tumour Necrosis Factor alpha
TRAIL-R2	Tumour necrosis factor-related Apoptosis-Inducing Ligand receptor 2
WBC	white blood count
