# Supplementary material for: IL-6, IL-1RA and Resistin as Predictors of Left Ventricular Remodelling and Major Adverse Cardiac Events in Patients with Acute ST Elevation Myocardial Infarction
Source: Diagnostics (Basel). 2022 Jan 21;12(2):266. doi: 10.3390/diagnostics12020266 (PMC8871243; doi:10.3390/diagnostics12020266)
Supplement: Supplementary file 1 [file diagnostics-12-00266-s001.zip › diagnostics-1542068-supplementary.pdf]

IL-6, IL-1RA and resistin as predictors of left ventricular remodelling and major adverse cardiac events in patients with acute ST elevation myocardial infarction

Supplementary materials

Table S1. Evolution of echocardiographic parameters in time (baseline vs 6 months follow) for the entire study population.

	Cohort at baseline	Cohort at follow up	P value
**2D LVEDV (ml)**	107.29±38.9	113.70±37.7	0.131
**2D LVESV (ml)**	66.41±35.45	63.89±29.1	0.740
**2D EF (%)**	39.85±8.9	46.2±8.85	**0.000**
**3D LVEDV (ml)**	114.63±33.37	124.43±32.26	**0.000**
**3D LVESV (ml)**	70.09±28.34	68.12±28.5	0.612
**3D LVEF (%)**	40.02±8.05	46.74±8.34	**0.000**
**LV GLS**	-12.44±4.17	-14.78±4.19	**0.000**
**LV mechanical dispersion**	65.94±24.4	62.53±20.9	0.340
**E/e′ (LV filling pressure)**	9.05±3.04	8.45±3.03	0.275

Table S2. Evolution of inflammatory markers in STEMI (baseline vs 6 months follow up).

	Baseline	Follow up	P value
**IL-6 (pg/ml)**	33.9±50.2	5.6±7.8	0.001
**IL1-RA (pg/ml)**	729.01±933.27	447.98±184.91	0.070
**Resistin (ng/ml)**	6.61±2.88	5.9±2.13	0.22

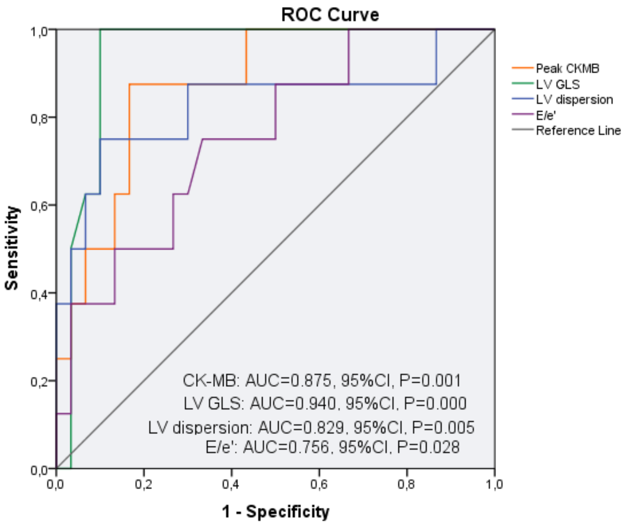

Figure S1. ROC curves for CK-MB, LV-GLS, LV dispersion and E/e' at admission for predicting LVR.

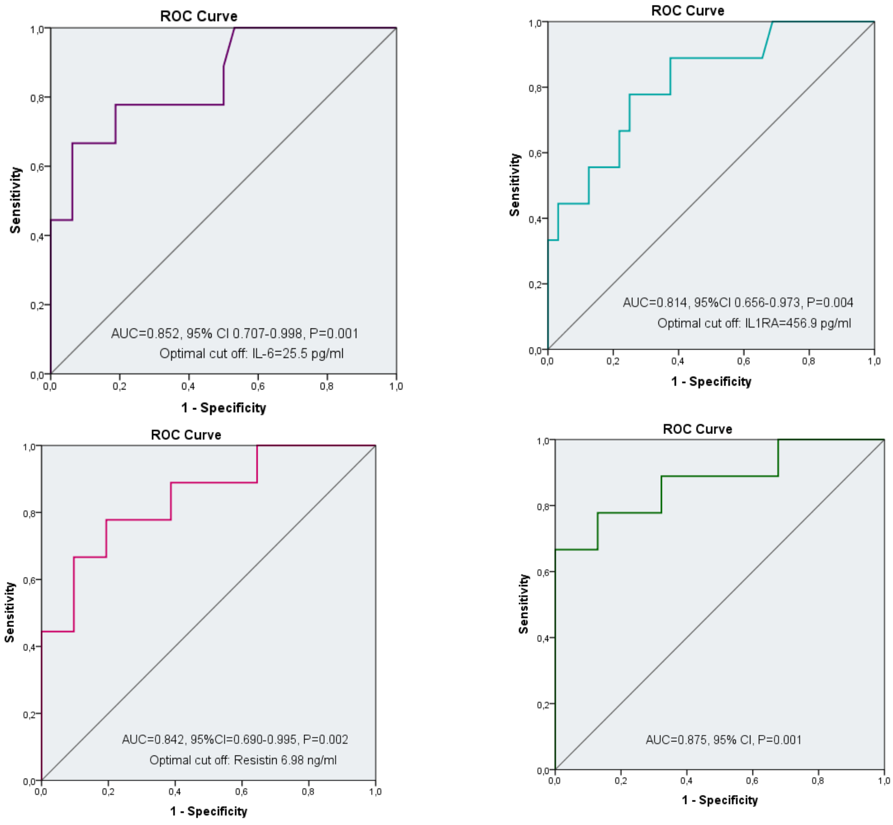

Figure S2. ROC curves of univariate variables (Il-6, IL-1RA, resistin) at admission for predicting MACE; The ROC curve for risk prediction model (simultaneously including the three cytokines) (bottom right).
